# The complete mitochondrial genome of the *Tonna galea* (Linnaeus, 1758) (Gastropoda: Tonnidae)

**DOI:** 10.1080/23802359.2025.2594294

**Published:** 2025-11-26

**Authors:** Dewei Cheng, Fangchao Zhu, Lintao Zhao, Xin Liu, Ying Qiao, Ersha Dang, Xuyang Chen

**Affiliations:** ^a^ Key Laboratory of Tropical Marine Ecosystem and Bioresource, Fourth Institute of Oceanography, Ministry of Natural Resources, Beihai, China; ^b^ Guangxi Key Laboratory of Beibu Gulf Marine Resources, Environment and Sustainable Development, Fourth Institute of Oceanography, Ministry of Natural Resources, Beihai, China; ^c^ Observation and Research Station of Coastal Wetland Ecosystem in Beibu Gulf, Ministry of Natural Resources, Beihai, China

**Keywords:** Mitogenome, phylogenetic analysis, Caenogastropoda

## Abstract

The complete mitochondrial genome of the giant tun shell, *Tonna galea* (Linnaeus, 1758), was sequenced using a hybrid Illumina and Nanopore approach. The assembled mitochondrial genome is a circular 17,504 bp molecule, encoding the typical 37 genes (13 PCGs, 22 tRNAs, and two rRNAs). Mitogenomic phylogenetic analysis robustly places *T. galea* as a sister taxon to *Tonna dolium* (Linnaeus, 1758). Additionally, the analysis strongly supports a close phylogenetic relationship between the families Tonnidae and Bursidae. These findings offer essential genetic insights into the phylogenetic framework of the family Tonnoidea.

## 1. Introduction

Mitochondrial genomes serve as powerful molecular markers for elucidating phylogenetic relationships and evolutionary processes in marine invertebrates (Dai et al. 2024; Lee et al. 2024; Matos et al. 2024). Within the diverse class Gastropoda, mitogenomic research on the family Tonnidae (Caenogastropoda) remains notably limited. Mitochondrial genomes of some tonnoid species (e.g. *Tonna sulcosa* Born, 1778; *Tonna dolium* Linnaeus, 1758) have been characterized (Zheng et al. 2023; Li et al. 2024), but the family Tonnidae itself is still underrepresented relative to other families within the superfamily.

The family Tonnidae includes *Tonna galea* (Linnaeus, 1758), commonly known as the giant tun shell, which represents an ecologically and economically significant species (de Simone and de Moluscos 1995; Rogelja and Lipej 2019). Despite its importance, the complete mitochondrial genome of *T. galea* has not been recorded to date. This lack of fundamental genetic data limits its utility for detailed comparative mitogenomic studies and precise phylogenetic placement within the Tonnoidea. Given the documented variations in gastropod mitochondrial genomes, including gene order rearrangements, nucleotide substitution rates, and strand compositional asymmetry (Li et al. 2024), a mitochondrial genome sequence for *T. galea* is needed to provide a reliable genetic foundation for evolutionary studies.

To address this gap, we sequenced, assembled, and annotated the complete mitochondrial genome of *T. galea* using a hybrid sequencing strategy combining Illumina short-read and Nanopore long-read technologies. This work provides a fundamental genomic resource that will facilitate future phylogenetic and evolutionary investigations into this species and the family Tonnidae.

## 2. Materials and methods

**Figure 1.**
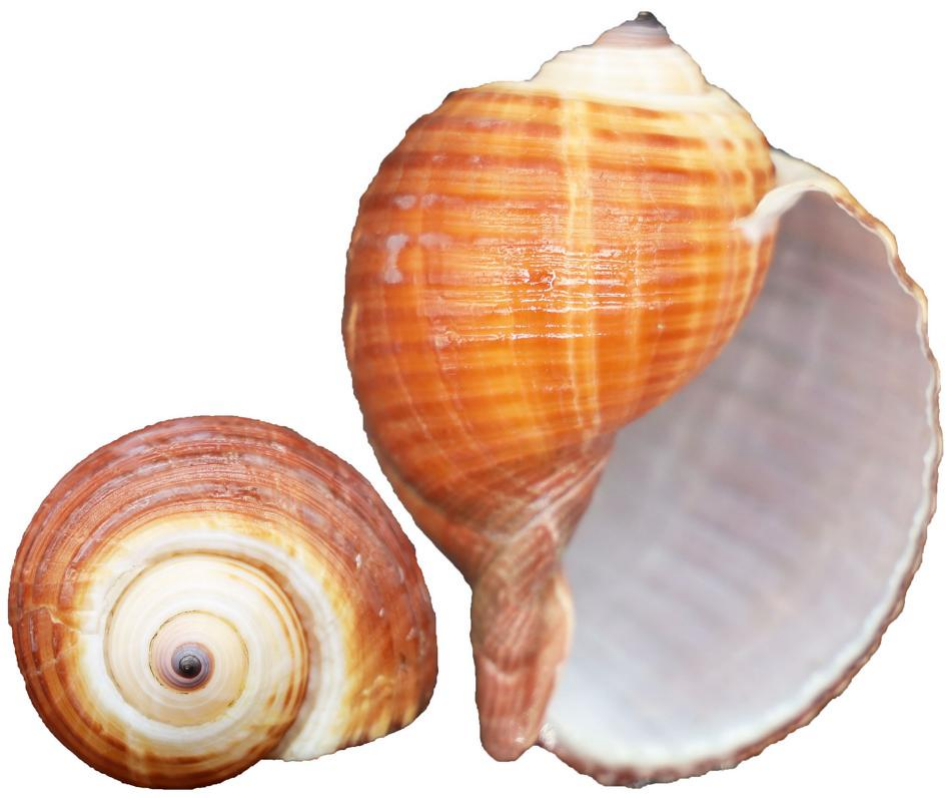
Specimen image of the *T. galea* was collected in April 2023 from Beibu Gulf, China (109°26′35.437092ʺ E; 21°3′16.475508ʺ N). The image was photographed by Dewei Cheng.

Specimens of *T. galea* were collected from the Beibu Gulf, China (coordinates: 109°26′35.437ʺ E, 21°3′16.475ʺ N) via commercial trawling (Figure 1). Muscle tissue was dissected and immediately preserved in 95% ethanol at the Laboratory of the Fourth Institute of Oceanography, Ministry of Natural Resources (voucher specimen number: TG20230423; contact: DW-Cheng, dwcheng2022@163.com). Total genomic DNA was extracted from the ethanol-preserved muscle tissue using the TIANamp Marine Animals DNA Kit (Tiangen, Beijing, China), strictly following the manufacturer’s protocol. DNA concentration and purity were precisely quantified using a Nanodrop 2000 spectrophotometer (Thermo Fisher Scientific, Waltham, MA). Dual-platform sequencing was performed using both the Nanopore PromethION (Oxford Nanopore Technologies, Oxford, UK) and the Illumina NovaSeq 6000 (Illumina, San Diego, CA) platform, with libraries constructed and sequenced on each system.

The mitochondrial genome was assembled *de novo* from the filtered clean reads. Initial assembly was performed using miniasm v0.3-r179 (Li 2016). The resulting assembly was subsequently polished using NextPolish v1.3.1 (https://github.com/Nextomics/NextPolish). Illumina clean reads were then aligned to the polished assembly using Bowtie2 v2.3.5.1 (Langmead and Salzberg 2012). The aligned Illumina reads and Nanopore reads were co-assembled using Unicycler v0.4.8 (Wick et al. 2017) to generate contigs. Sequencing depth and coverage are shown in Supplementary material Figure S1. Subsequently, the mitochondrial genome was annotated. Initial gene prediction was performed with MITOS2 (Donath et al. 2019) and MitoZ (Meng et al. 2019). The results were integrated and manually curated in Geneious Prime v2021.0.3, where protein-coding gene (PCG) boundaries were verified via open reading frame analysis. Finally, the circular nature and integrity of the final *T. galea* mitochondrial genome assembly were confirmed and visualized using Proksee (Grant et al. 2023), and its skewness was assessed using the equations: AT skew = (A − T)/(A + T); GC skew = (G − C)/(G + C) (Ke et al. 2023).

A maximum-likelihood phylogenetic tree was reconstructed following the methodology described by Yang et al. (2025) using PhyloSuite v1.2.3 (Zhang et al. 2020). The analysis included the concatenated sequences of 13 mitochondrial PCGs from 26 species (including the *T. galea* mitochondrial genome obtained in this study). The genus *Conus* (*Conus consors* and *Conus quercinus*) was designated as the outgroup, a choice consistent with previous phylogenetic studies on Tonnoidea and based on its established taxonomic distance as a member of the closely related superfamily Conoidea (Zheng et al. 2023). Within the PhyloSuite environment, each PCG was individually aligned using the implemented MAFFT v7.471 (Katoh and Standley 2013) module, and the resulting alignments were subsequently trimmed with trimAl v1.2 (Capella-Gutiérrez et al. 2009). The best-fit partition scheme and substitution models were selected under the Bayesian information criterion (BIC) using ModelFinder (Kalyaanamoorthy et al. 2017). The final tree was inferred with IQ-TREE v2.2.0 (Nguyen et al. 2015) under the edge-linked partition model, with branch support assessed from 5000 ultrafast bootstrap replicates. The resulting tree was visualized and annotated using the online tool iTOL v7 (Letunic and Bork 2007).

## 3. Results

**Figure 2.**
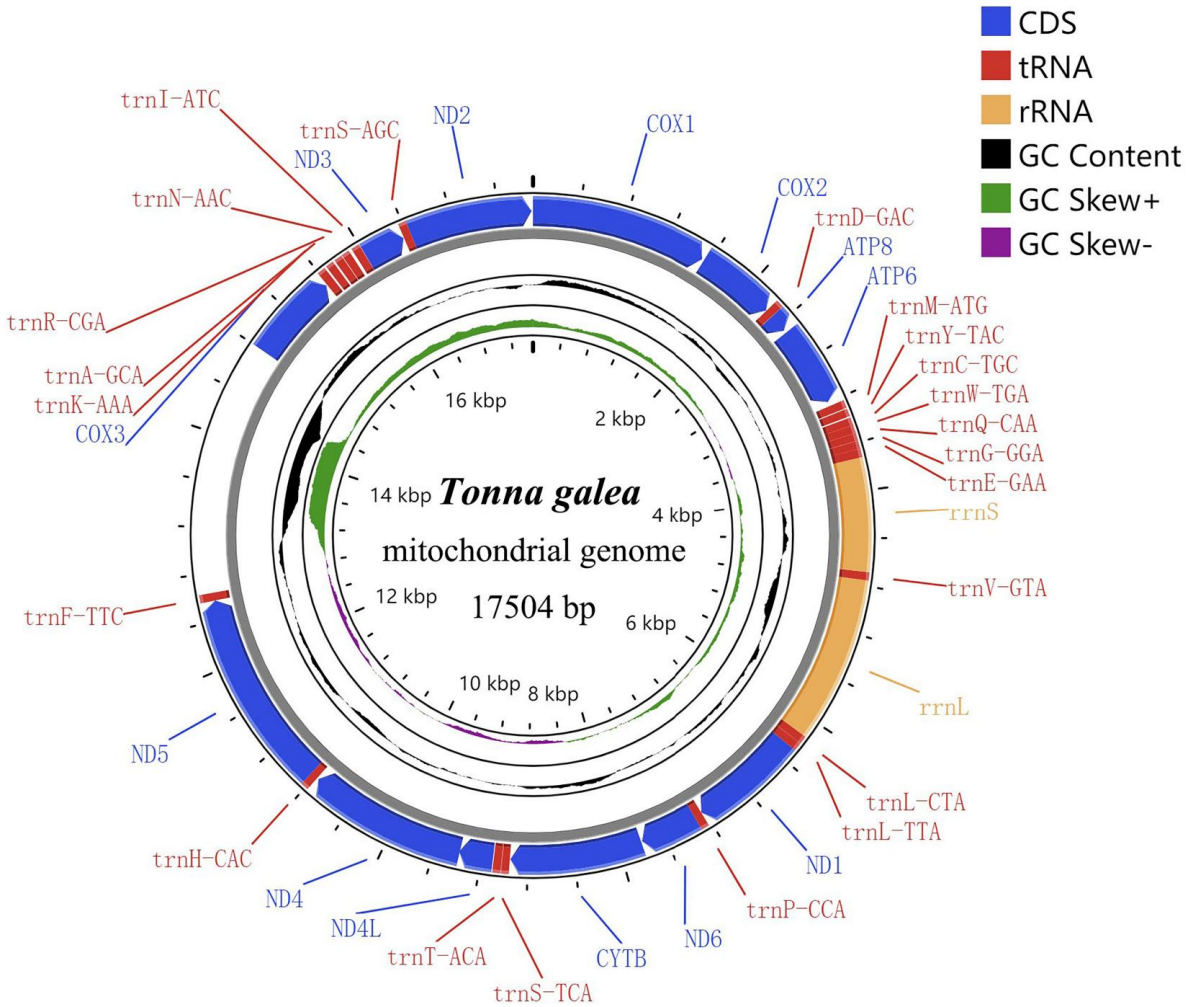
Mitochondrial genome map of *T. galea* (GenBank accession number: OR282483). Genes are color-coded according to their functional classification. Blue represents 13 PCGs, red represents 23 tRNAs, and yellow represents two rRNAs. The black color in the inner ring indicates GC content, while the green and purple represent GC skew.

The complete mitochondrial genome of *T. galea* was sequenced and assembled into a circular DNA molecule of 17,504 bp (NCBI accession no. OR282483). The genome contains the standard 37 mitochondrial genes: 13 PCGs, 22 transfer RNA (tRNA) genes, and two ribosomal RNA (rRNA) genes, with gene order identical to other Tonnoidea species (Figure 2). The base composition analysis revealed a high A + T content of 72.5% (A: 32.4%, T: 40.1%, G: 15.2%, C: 12.3%), with strand asymmetry evidenced by a negative AT skew (−0.106) and positive GC skew (0.106). As detailed in Table 1, all PCGs used ATG as the start codon, while most terminated with TAA except for ND1, ND4L, and COX3, which used TAG as stop codon. Of the 22 tRNA genes, eight are encoded on the light strand. Their lengths range from 65 to 72 bp. The 12S (958 bp) and 16S rRNA (1396 bp) genes are separated by trnV-GTA.

**Table 1.** Characteristics of the mitochondrial genome of *T. galea.*

Start	End	Length	Strand	Initial codons	Terminal codons	Gene
1	1536	1536	H	ATG	TAA	COX1
1554	2240	687	H	ATG	TAA	COX2
2245	2312	68	H			trnD-GAC
2314	2472	159	H	ATG	TAA	ATP8
2498	3193	696	H	ATG	TAA	ATP6
3232	3299	68	L			trnM-ATG
3308	3373	66	L			trnY-TAC
3385	3450	66	L			trnC-TGC
3451	3518	68	L			trnW-TGA
3519	3583	65	L			trnQ-CAA
3586	3652	67	L			trnG-GGA
3653	3723	71	L			trnE-GAA
3724	4681	958	H			rrnS
4682	4750	69	H			trnV-GTA
4751	6146	1396	H			rrnL
6147	6215	69	H			trnL-CTA
6217	6285	69	H			trnL-TTA
6286	7227	942	H	ATG	TAG	ND1
7228	7296	69	H			trnP-CCA
7298	7798	501	H	ATG	TAA	ND6
7808	8947	1140	H	ATG	TAA	CYTB
8953	9017	65	H			trnS-TCA
9022	9089	68	L			trnT-ACA
9098	9394	297	H	ATG	TAG	ND4L
9388	10764	1377	H	ATG	TAA	ND4
10779	10843	65	H			trnH-CAC
10844	12565	1722	H	ATG	TAA	ND5
12566	12633	68	H			trnF-TTC
14801	15580	780	H	ATG	TAG	COX3
15590	15661	72	H			trnK-AAA
15679	15746	68	H			trnA-GCA
15764	15832	69	H			trnR-CGA
15837	15904	68	H			trnN-AAC
15932	16002	71	H			trnI-ATC
16006	16359	354	H	ATG	TAA	ND3
16366	16433	68	H			trnS-AGC
16434	17492	1059	H	ATG	TAA	ND2

**Figure 3.**
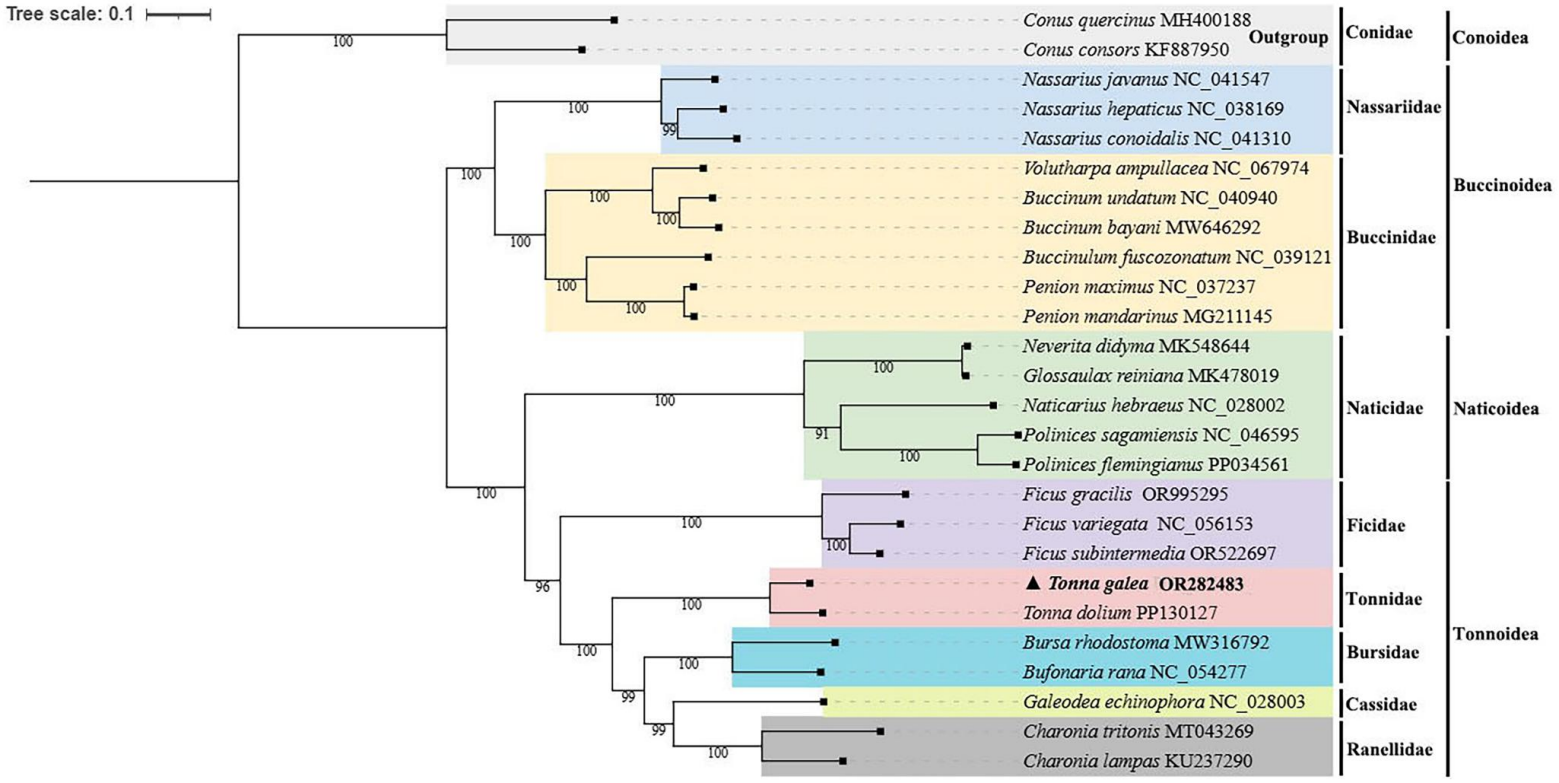
Maximum-likelihood phylogenetic tree based on sequences from 13 PCGs of the mitochondrial genome. Bootstrap values are indicated on the branches. All species in the tree are labeled with their scientific names and NCBI GenBank accession numbers on the right side. The position of *T. galea* was highlighted by a black triangle and bold. The following sequences were used: *Tonna galea* (OR282483, in this study), *Conus quercinus* (MH400188, Chen et al. 2018), *Conus consors* (KF887950, Brauer et al. 2012), *Nassarius hepaticus* (NC_038169, Yang, Ye, et al. 2019), *Nassarius javanus* (NC_041547, Yang, Li, et al. 2019), *Nassarius conoidalis* (NC_041310, Yang, Li, et al. 2019), *Volutharpa ampullacea* (NC_067974, Qu et al. 2024), *Buccinulum fuscozonatum* (NC_039121,Vaux et al. 2018), *Buccinum bayani* (MW646292, Kim et al. 2022), *Buccinum undatum* (NC_040940, Jónsson et al. 2019), *Penion maximus* (NC_037237, unpublished), *Penion mandarinus* (MG211145, unpublished), *Neverita didyma* (MK548644, Wang et al. 2019), *Glossaulax reiniana* (MK478019, Liu et al. 2020), *Naticarius hebraeus* (NC_028002, Osca et al. 2015), *Polinices sagamiensis* (NC_046595, Liu et al. 2020), *Polinices flemingianus* (PP034561, Li et al. 2024), *Ficus gracilis* (OR995295, Li et al. 2024), *Ficus variegata* (NC_056153, Wang et al. 2021), *Ficus subintermedia* (OR522697, unpublished), *Tonna dolium* (PP130127, Li et al. 2024), *Bursa rhodostoma* (MW316792, Sanders et al. 2021), *Bufonaria rana* (NC_054277, Zhong et al. 2020), *Galeodea echinophora* (NC_028003, Osca et al. 2015), *Charonia tritonis* (MT043269, Unpublished), and *Charonia lampas* (KU237290, Choi and Hwang 2021).

Phylogenetic reconstruction based on the concatenated sequences of 13 PCGs from 26 species robustly supported the sister relationship between *T. galea* and *T. dolium* (bootstrap value = 100%). Importantly, the Tonnidae clade showed its closest relationship to Bursidae, represented by *Bursa rhodostoma* Sowerby, 1835 and *Bufonaria rana* Linnaeus, 1758, with strong support, as clearly shown in the phylogenetic tree (Figure 3). This well-resolved topology provides reliable insights into the phylogenetic position of *T. galea* within the Tonnoidea superfamily.

## 4. Discussion and conclusions

This study presents the first complete mitochondrial genome of *T. galea*, a significant step toward addressing the underrepresentation of tonnid mitogenomes in public databases. The newly characterized mitogenome of 17,504 bp exhibits a typical gene order that is highly conserved within the Tonnoidea superfamily, suggesting a stable genomic architecture in this group (Osca et al. 2015). The high A + T content (72.5%) and the observed strand asymmetry are consistent with compositional biases previously documented in other gastropod mitogenomes (Zhong et al. 2020; Choi and Hwang 2021; Wang et al. 2021). The phylogenetic reconstruction provides robust mitogenomic evidence for the evolutionary relationships within caenogastropods. It strongly supports a sister-species relationship between *T. galea* and *T. dolium*, clarifying their close affiliation within the genus *Tonna*. Furthermore, the analysis confirms a close phylogenetic relationship between Tonnidae and Bursidae, represented by *B. rhodostoma* and *B. rana*. This finding is congruent with a previous study by Zheng et al. (2023), thereby validating and strengthening this specific phylogenetic inference.

In conclusion, our study establishes *T. galea* as a phylogenetically conserved member of the Tonnoidea superfamily, characterized by conserved gene order and close evolutionary ties to Bursidae. These findings provide a solid foundation for future studies on gastropod evolution, molecular ecology, and conservation genomics of marine mollusks.

## Supplementary Material

Figure S1 Read coverage depth map.jpg

## Data Availability

The genome sequence data that support the findings of this study are openly available in GenBank of NCBI at https://www.ncbi.nlm.nih.gov/ under the accession no. OR282483. The associated BioProject, SRA, and BioSample numbers are PRJNA1194572, SRR31869525–SRR33694971, and SAMN45190311, respectively.

## References

[CIT0001] Brauer A et al. 2012. The mitochondrial genome of the venomous cone snail *Conus consors*. PLoS One. 7(12):e51528. 10.1371/journal.pone.0051528 23236512 PMC3517553

[CIT0002] Capella-Gutiérrez S, Silla-Martínez JM, Gabaldón T. 2009. trimAl: a tool for automated alignment trimming in large-scale phylogenetic analyses. Bioinformatics. 25(15):1972–1973. 10.1093/bioinformatics/btp348 19505945 PMC2712344

[CIT0003] Chen PW, Wu WL, Hwang DF. 2018. The complete mitochondrial genome of *Conus quercinus* (Neogastropoda: Conidae). Mitochondrial DNA B Resour. 3(2):933–934. 10.1080/23802359.2018.1501314 33490546 PMC7800317

[CIT0004] Choi EH, Hwang UW. 2021. The complete mitochondrial genome of an endangered triton snail *Charonia lampas* (Littorinimorpha: Charoniidae) from South Korea. Mitochondrial DNA B Resour. 6(3):956–958. 10.1080/23802359.2021.1889416 33796697 PMC7995882

[CIT0005] Dai M et al. 2024. The complete mitochondrial genome of *Mya japonica* (Jay, 1857 Myida: Myidae). Mitochondrial DNA B Resour. 9(8):991–994. 10.1080/23802359.2024.2387247 39108544 PMC11302462

[CIT0006] de Simone L, de Moluscos S. 1995. Anatomical study on *Tonna galea* (Linne, 1758) and *Tonna maculosa* (Dillwyn, 1817) (Mesogastropoda, Tonnoidea, Tonnidae) from Brazilian Region. Malacologia. 37(1):23–32.

[CIT0007] Donath A et al. 2019. Improved annotation of protein-coding genes boundaries in metazoan mitochondrial genomes. Nucleic Acids Res. 47(20):10543–10552. 10.1093/nar/gkz833 31584075 PMC6847864

[CIT0008] Grant JR et al. 2023. Proksee: in-depth characterization and visualization of bacterial genomes. Nucleic Acids Res. 51(W1):W484–W492. 10.1093/nar/gkad326 37140037 PMC10320063

[CIT0009] Jónsson ZO et al. 2019. The mitochondrial genome of common whelk *Buccinum undatum* (Neogastropoda: Buccinidae). Mitochondrial DNA Part B. 4(1):457–459. 10.1080/23802359.2018.1545534

[CIT0010] Kalyaanamoorthy S, Minh BQ, Wong TKF, von Haeseler A, Jermiin LS. 2017. ModelFinder: fast model selection for accurate phylogenetic estimates. Nat Methods. 14(6):587–589. 10.1038/nmeth.4285 28481363 PMC5453245

[CIT0011] Katoh K, Standley DM. 2013. MAFFT multiple sequence alignment software version 7: improvements in performance and usability. Mol Biol Evol. 30(4):772–780. 10.1093/molbev/mst010 23329690 PMC3603318

[CIT0012] Ke ZL et al. 2023. Characterization of the complete mitochondrial genome of the elongate loach and its phylogenetic implications in Cobitidae. Animals. 13(24):3841. 10.3390/ani13243841 38136877 PMC10740543

[CIT0013] Kim KR, Han HS, Kim YH, Park JY, Bang IC. 2022. The complete mitochondrial genome of *Buccinum bayani* (Gastropoda: Buccininae) in Korea. Mitochondrial DNA B Resour. 7(6):916–917. 10.1080/23802359.2022.2079098 35692638 PMC9176358

[CIT0014] Langmead B, Salzberg SL. 2012. Fast gapped-read alignment with bowtie 2. Nat Methods. 9(4):357–359. 10.1038/nmeth.1923 22388286 PMC3322381

[CIT0015] Lee Y, Kim KB, Choi EH, Hwang UW. 2024. Complete mitochondrial genome of the worm snail *Thylacodes adamsii* (Littorinimorpha: Vermetidae) from South Korea. Mitochondrial DNA B Resour. 9(6):753–757. 10.1080/23802359.2024.2368209 38895513 PMC11185085

[CIT0016] Letunic I, Bork P. 2007. Interactive Tree Of Life (iTOL): an online tool for phylogenetic tree display and annotation. Bioinformatics. 23(1):127–128. 10.1093/bioinformatics/btl529 17050570

[CIT0017] Li F et al. 2024. The molecular phylogeny of Caenogastropoda (Mollusca, Gastropoda) based on mitochondrial genomes and nuclear genes. Gene. 928:148790. 10.1016/j.gene.2024.148790 39053659

[CIT0018] Li H. 2016. Minimap and miniasm: fast mapping and de novo assembly for noisy long sequences. Bioinformatics. 32(14):2103–2110. 10.1093/bioinformatics/Btw152 27153593 PMC4937194

[CIT0019] Liu H, Yang Y, Sun SE, Kong L, Li Q. 2020. Mitogenomic phylogeny of the Naticidae (Gastropoda: Littorinimorpha) reveals monophyly of the Polinicinae. Zool Scr. 49(3):295–306. 10.1111/zsc.12412

[CIT0020] Matos A et al. 2024. The complete female mitogenome of *Potomida semirugata* (Lamarck, 1819). Mitochondrial DNA B Resour. 9(7):892–896. 10.1080/23802359.2024.2378964 39027116 PMC11257011

[CIT0021] Meng G, Li Y, Yang C, Liu S. 2019. MitoZ: a toolkit for animal mitochondrial genome assembly, annotation and visualization. Nucleic Acids Res. 47(11):e63. 10.1093/nar/gkz173 30864657 PMC6582343

[CIT0022] Nguyen LT, Schmidt HA, von Haeseler A, Minh BQ. 2015. IQ-TREE: a fast and effective stochastic algorithm for estimating maximum-likelihood phylogenies. Mol Biol Evol. 32(1):268–274. 10.1093/molbev/msu300 25371430 PMC4271533

[CIT0023] Osca D, Templado J, Zardoya R. 2015. Caenogastropod mitogenomics. Mol Phylogenet Evol. 93:118–128. 10.1016/j.ympev.2015.07.011 26220836

[CIT0024] Qu J et al. 2024. Gene characterization and phylogenetic analysis of four mitochondrial genomes in Caenogastropoda. Acta Oceanol Sin. 43(2):137–150. 10.1007/s13131-023-2258-7

[CIT0025] Rogelja M, Lipej L. 2019. Occurrence of Giant Tun, *Tonna galea* (Linnaeus, 1758) (Gastropoda: Tonnidae) in the marine waters off Slovenia (Northern Adriatic Sea). Ann Ser Hist Nat Sci Res Cent Repub Slov. 29(1):121–124. 10.19233/ASHN.2019.12

[CIT0026] Sanders MT, Merle D, Laurin M, Bonillo C, Puillandre N. 2021. Raising names from the dead: a time-calibrated phylogeny of frog shells (Bursidae, Tonnoidea, Gastropoda) using mitogenomic data. Mol Phylogenet Evol. 156:107040. 10.1016/j.ympev.2020.107040 33310060

[CIT0027] Vaux F et al. 2018. Evolutionary lineages of marine snails identified using molecular phylogenetics and geometric morphometric analysis of shells. Mol Phylogenet Evol. 127:626–637. 10.1016/j.ympev.2018.06.009 29913310

[CIT0028] Wang Q et al. 2021. Characterization of the complete mitochondrial genome of *Ficus variegata* (Littorinimorpha: Ficidae) and molecular phylogeny of Caenogastropoda. Mitochondrial DNA B Resour. 6(3):1126–1128. 10.1080/23802359.2021.1901628 33796763 PMC7995913

[CIT0029] Wang Z et al. 2019. The mitochondrial genome of the marine gastropod *Neverita didyma* (Roding, 1798) (Mollusca: Gastropoda). Mitochondrial DNA Part B. 4(1):1545–1546. 10.1080/23802359.2019.1601535

[CIT0030] Wick RR, Judd LM, Gorrie CL, Holt KE. 2017. Unicycler: resolving bacterial genome assemblies from short and long sequencing reads. PLoS Comput Biol. 13(6):e1005595. 10.1371/journal.pcbi.1005595 28594827 PMC5481147

[CIT0031] Yang H, Ye Y, Liu S, Xu M, Guo B. 2019. Characterization of complete mitochondrial genome of *Nassarius hepaticus* (Stenoglossa, Nassariidae). Mitochondrial DNA Part B. 4(1):446–447. 10.1080/23802359.2018.1555016

[CIT0032] Yang Y et al. 2025. The complete mitochondrial genome of the snapping shrimp, *Alpheus brevicristatus* De Haan, 1844 (Crustacea, Decapoda, Alpheidae). Mitochondrial DNA B Resour. 10(7):554–557. 10.1080/23802359.2025.2487072 40503060 PMC12152993

[CIT0033] Yang Y, Li Q, Kong L, Yu H. 2019. Mitogenomic phylogeny of *Nassarius* (Gastropoda: Neogastropoda). Zool Scr. 48(3):302–312. 10.1111/zsc.12343

[CIT0034] Zhang D et al. 2020. PhyloSuite: an integrated and scalable desktop platform for stream-lined molecular sequence data management and evolutionary phylogenetics studies. Mol Ecol Resour. 20(1):348–355. 10.1111/1755-0998.13096 31599058

[CIT0035] Zheng J et al. 2023. Mitogenomic phylogeny of Tonnoidea Suter, 1913 (1825) (Gastropoda: Caenogastropoda). Animals. 13(21):3342. 10.3390/ani13213342 37958096 PMC10649890

[CIT0036] Zhong S, Liu Y, Huang G, Huang L. 2020. The first complete mitochondrial genome of Bursidae from *Bufonaria rana* (Caenogastropoda: Tonnoidea). Mitochondrial DNA Part B. 5(3):2585–2586. 10.1080/23802359.2020.1781575

