# Supplementary figures and images for: The complete mitochondrial genome of the Tonna galea (Linnaeus, 1758) (Gastropoda: Tonnidae)
Source: Mitochondrial DNA B Resour. 2025 Nov 26;10(12):1271–5. doi: 10.1080/23802359.2025.2594294 (PMC12667315; doi:10.1080/23802359.2025.2594294)

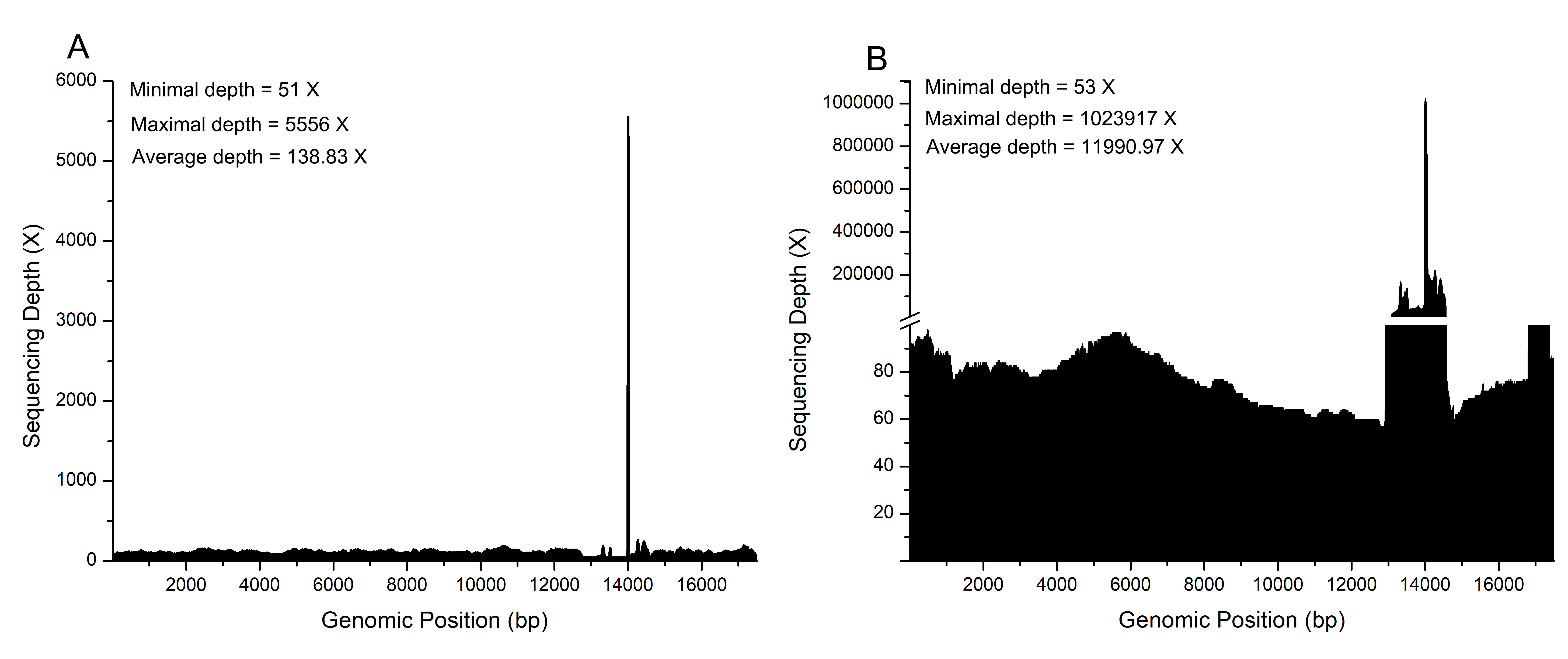

Supplement: Figure S1 Read coverage depth map.jpg [file TMDN_A_2594294_SM7701.jpg]
